# Interleukin-33 / Cyclin D1 imbalance in severe liver steatosis predicts susceptibility to ischemia reperfusion injury

**DOI:** 10.1371/journal.pone.0216242

**Published:** 2019-04-29

**Authors:** Kelley G. Núñez, Anderson Frank, Janet Gonzalez-Rosario, Gretchen Galliano, Kim Bridle, Darrell Crawford, John Seal, Frank Abbruscato, Himanshu Vashistha, Paul T. Thevenot, Ari J. Cohen

**Affiliations:** 1 Institute of Translational Research, Ochsner Health System, New Orleans, Louisiana, United States of America; 2 Pathology, Ochsner Health System, New Orleans, Louisiana, United States of America; 3 Gallipoli Medical Research Institute and Faculty of Medicine, The University of Queensland School of Medicine, Greenslopes, Brisbane, Australia; 4 Multi-organ Transplant Center, Ochsner Health System, New Orleans, Louisiana, United States of America; Indiana University, UNITED STATES

## Abstract

Transplanting donor livers with severe macrosteatosis is associated with increased risk of primary non-function (PNF). The purpose of this study was to identify steatosis-driven biomarkers as a predisposition to severe liver damage and delayed recovery following ischemia reperfusion injury. Wistar rats were fed a methionine- and choline-deficient (MCD) diet for up to three weeks to achieve severe macrosteatosis (>90%). Animals underwent diet withdrawal to control chow and/or underwent ischemia reperfusion and partial hepatectomy injury (I/R-PHx) and reperfused out to 7 days on control chow. For animals with severe macrosteatosis, hepatic levels of IL-33 decreased while Cyclin D1 levels increased in the absence of NF-κB p65 phosphorylation. Animals with high levels of nuclear Cyclin D1 prior to I/R-PHx either did not survive or had persistent macrosteatosis after 7 days on control chow. Survival 7 days after I/R-PHx fell to 57% which correlated with increased Cyclin D1 and decreased liver IL-33 levels. In the absence of I/R-PHx, withdrawing the MCD diet normalized IL-33, Cyclin D1 levels, and I/R-PHx survival back to baseline. In transplanted grafts with macrosteatosis, higher Cyclin D1 mRNA expression was observed. Shifts in Cyclin D1 and IL-33 expression may identify severely macrosteatotic livers with increased failure risk if subjected to I/R injury. Clinical validation of the panel in donor grafts with macrosteatosis revealed increased Cyclin D1 expression corresponding to delayed graft function. This pre-surgical biomarker panel may identify the subset of livers with increased susceptibility to PNF.

## Introduction

Despite 7,127 liver transplants being performed in the United States in 2015, the number of patients awaiting liver transplantation increased to over 14,000 patients[1]. Although waitlist mortality (1,600 patients) and dropout (1,200 patients) have stabilized from a trend of annual increases, donor shortage remains a significant issue. Additionally, decreased donor liver quality continues to influence utilization, and is estimated to result in a decrease donor utilization rate from 78% to 44% by the year 2030. To offset this imbalance, grafts with high DRI (donor risk index) are with known risk factors such as advanced age, with long cold ischemic time, or steatosis and have a high risk of PNF[2–5].

Hepatic steatosis results from the imbalanced triglyceride import and/or de novo synthesis versus the rate of export or consumption. Donor steatosis has been estimated in 13–30% of procurements[3], and is expected to significantly rise due to the obesity epidemic and increasing rates of nonalcoholic fatty liver disease (NAFLD).

Steatotic livers are graded as mild macrosteatosis (<30%), moderate (30–60%), or severe (>60%) based on the percentage of hepatocytes containing fat droplets. Safe utilization of donor livers with moderate to severe macrosteatosis remains controversial, partially due to inaccurate estimation of donor steatosis leading to inconsistent patient outcomes[6].

Simple steatosis, the initial pathological stage of NAFLD is of clinical relevance to high DRI grafts for liver transplantation. The molecular mechanisms triggering simple steatosis and progression to NAFLD are rapidly emerging although strategies to oppose progression to nonalcoholic steatohepatitis (NASH) remain elusive. Steatosis-driven biomarkers that can predict graft failure risk may help increase safe utilization.

In this study, we recreated an animal model of escalating simple steatosis aiming to identify steatosis-driven biomarkers of dysfunction to predict liver damage following I/R injury. This study provides insight into predictors of PNF in steatotic grafts to refine the selection of suitable steatotic grafts for transplantation.

## Materials and methods

### Chemical reagents and antibodies

p-NF-κB [93H1], NFκB [D14E12], p-Akt Ser473 [D9E], Akt (pan) [C67E7], Cyclin D1 [92G2], p-Rb [D59B7], Rb [D20], GSK-3β [27C10], Caspase-3 [8G10], cleaved Caspase-3 [5A1E] from Cell Signaling, Danvers, MA; IL-33 (M-266) from Santa Cruz Biotechnology, Dallas, TX; GAPDH [GAPDH-71.1], Actin [A2228], Vinculin [hVIN-1] from Sigma Aldrich, St. Louis, MO; p-GSK-3β [75745] and nuclear matrix protein p84 [EPR5662(2)] from Abcam, Cambridge, MA. Immunohistochemical antibodies used were as follows: CD163 [EPR19518] from Abcam, Cambridge, MA and CD3 [1F4] from BD Biosciences, San Jose, CA.

### Animal models of steatosis and I/R-PHx

All animal experiments were in accordance with institutional guidelines and comply with criteria outlined by the Guide for the Care and Use of Laboratory Animals. The protocol was approved by the Ochsner Institutional Animal Care and Use Committee (Protocol Number: 2015–16. All surgeries were performed under isoflurane anesthesia, and all efforts made to minimize suffering. All animal surgeries were performed by two staff members trained in rat hepatic I/R surgeries, with each experiment reproduced independently by both surgeons.

Male Wistar rats (~125 grams, 4 weeks of age, Charles River Laboratories, Wilmington, MA) were maintained in cages with corn cob bedding. Rats were acclimated for 1 week and transitioned to a MCD diet (TD.90262, Envigo), or control chow (TD.94149, Envigo). Rats were fed experimental diets for weekly intervals ranging from 1 to 3 weeks. In diet reversal experiments, rats were fed the MCD diet for the indicated period of time, then switched to control chow for either 24 hours or 7 days. All experimental endpoints (dietary, withdrawal, and ischemia) contained 3 animals and was performed in duplicate. For survival experiments involving I/R-PHx, each endpoint contained 4 animals with exception to 3 week endpoints which contained 15 animals. All animals were fasted overnight before humane endpoint.

I/R-PHx injury was performed as previously published[7] with slight modifications. Briefly, a midline laparotomy was performed to exposure the liver. Partial (70%) liver ischemia was induced by placing a microvascular clamp on the portal triad supplying the left lateral and median lobes for 60 minutes. Prior to clamp removal, 30% partial hepatectomy was performed by resecting the non-ischemic liver lobes.

Human endpoint criteria were defined and applied for all experiments involving animal survival after I/R-PHx. Survival endpoint was declared if animals failed to right 6 hours post-surgery, exhibited signs of distress 24 hours post-surgery (defined as decreased activity, failure to drink, or consume moistened chow placed within the cage), or found deceased at routine monitoring intervals. All surgeries were performed between the hours of 0600 and 0900. Animals were monitored 6 hours post-surgery or until righted and returning to normal activity. Afterward, animals were monitored every 3 hours from 0600 to 1800 hours. Survival studies were carried out for 7 days post-surgery. Any animals meeting endpoint criteria were immediately euthanized. A total of 16 animals were monitored in survival studies, with 12 animals requiring euthanasia, and 4 found deceased. In all instances, pathology suggested liver sinusoidal congestion leading to liver failure as cause of death.

Biopsies were performed immediately prior to ischemia by cutting a wedge (~0.2 grams) from the left lateral lobe. Absorbable hemostat tape was placed over area to promote hemostasis.

### Histology

Livers were processed to formalin-fixed paraffin embedded blocks and sections (5μm) were prepared for hematoxylin and eosin, trichrome (Sigma), and immunohistochemistry staining. The percentage of total micro- and macrosteatosis was quantified by a blinded hepatobiliary pathologist. Antibodies used for immunohistochemistry.

### Serum and hepatic ELISA

Serum was prepared from blood obtained from the inferior vena cava prior to humane endpoint. For hepatic lysate ELISA, the left lobe was minced and homogenized in lysis buffer containing 0.5% Triton-X 100. Serum and/or hepatic lysate normalized to protein content were assayed for IL-33, IL-1α, TNF-α, and IL-6 (R&D Systems, Minneapolis, MN) and HMGB1 (Antibodies-online, Atlanta, GA).

### Triglycerides and alanine aminotransaminase (ALT) assay

Total liver triglycerides were quantified in lysate prepared from equal weight of minced left lobe according to manufactures instructions (Cayman Chemical, Ann Arbor, MI). Serum ALT concentration was determined in serum according to manufactures instructions (Cayman Chemical).

### Western blot

For non-phosphorylated targets, lysate was prepared in Nonidet P-40 substitute buffer. For phosphorylated targets, lysate was prepared in 1% Triton-X 100 buffer. Nuclear and cytoplasmic fractions of the median lobe were prepared using the NU-PER fractioning kit (ThermoFisher Scientific, Waltham, MA). All blots were run on 12% or 4–20% Novex pre-cast Tris-Glycine gels and transferred to PVDF membranes using the iBlot 2 (ThermoFisher Scientific). Membrane blocking, primary, and secondary antibody staining were performed according to antibody manufacturers’ recommendation. Blots were developed with ECL or SuperSignal West Pico reagent (ThermoFisher Scientific).

### RNA extraction and quantitative PCR

Liver tissue was weighed, homogenized in TRIzol Reagent (ThermoFisher Scientific) and extracted following manufacturer protocol. QuantiNova Reverse Transcription kit (Qiagen, San Diego, CA) was used to convert RNA to complementary DNA. TaqMan probes (ThermoFisher Scientific) were used for Cyclin D1 (CCND1) [human, Hs00765553_m1; rat, Rn00432359_m1], and GAPDH [human, Hs03929097_g1; rat, Rn01775763_g1] and performed on a BioRad CFX 384 machine (Hercules, CA). Quantitative PCR data is presented as normalized expression (ΔΔCt) using GAPDH as a reference gene. Normalized expression was calculated in two steps using the following equations:

$${\text{Ct}}_{\text{GAPDH}}{-\text{Ct}}_{\text{CCND1}}=\Delta \text{Ct}$$ (1)

$$2-{}^{\left(\Delta \text{CtSample}-\text{Average}(\Delta \text{CtControl})\right)}=\Delta \Delta \text{Ct}$$ (2)

### Donor livers

Biopsies (~50mg) from nine donor livers (*Homo sapiens)* used for transplantation were collected and banked at our institution from 2015–2016. The percentage of total macro- and microsteatosis was determined using hematoxylin and eosin stains by a blinded hepatobiliary pathologist. Donor demographics and aspartate aminotransferase (AST) levels from the recipient 24 hours after transplantation were extracted from the medical record.

### Statistical analyses

Statistical analyses were performed using Prism 7 software (GraphPad Software, San Diego, CA). One-way ANOVA, with post hoc Tukey-Kramer test, was used to determine significance between the means of groups. One-way ANOVA, with post hoc Dunnett test, was performed to compare control and MCD fed animals. The data are reported as the mean (± standard error of the mean) calculated by GraphPad Prism 7.

## Results

### Simple steatosis animal model and steatosis-driven biomarkers to predict liver failure

**Fig 1:**
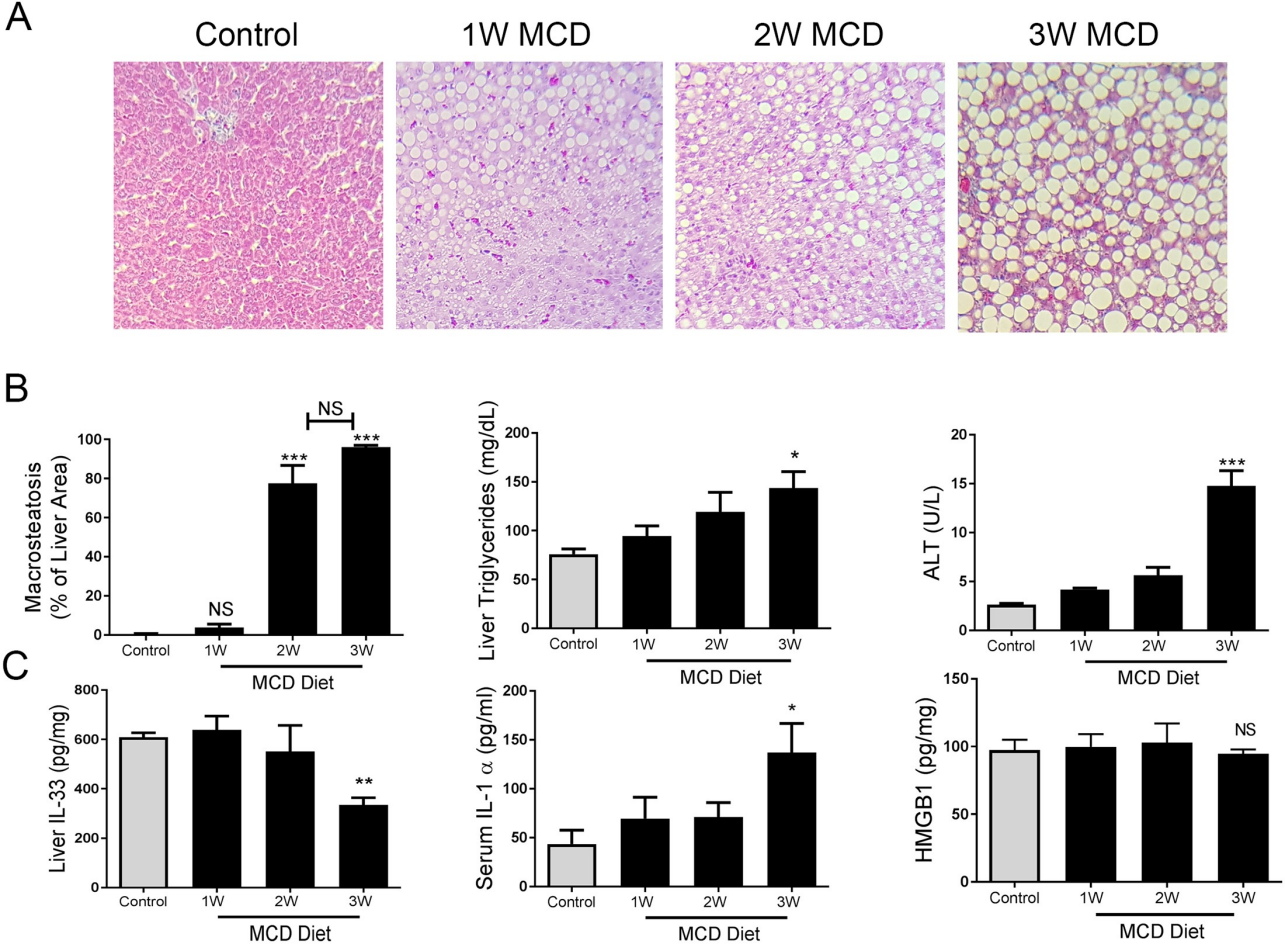
Rat model of diet-induced progressive simple steatosis. (A) Trichrome stains of representative rats on MCD diet for 1, 2, and 3 weeks. (B) Percentage of macrosteatosis (***p<0.0001), liver triglycerides (*p<0.03), and serum alanine aminotransferase levels (**p<0.001) at each diet endpoint. (C) Liver IL-33 (*p<0.02), serum IL-1α levels (*p<0.05) and HMGB1 levels (**p<0.001) in each MCD diet endpoint.

Donor livers often contain a mix of micro- and macrosteatosis. To establish an animal model that mimicked donor steatosis, rats were fed the MCD diet. Histological examination showed animals fed MCD diet closely resembled donor steatosis compared (Fig 1A). MCD feeding periods were optimized to generate distinct grades of macrosteatosis (MaS) and characterized at weekly endpoints. Rats were fed a MCD diet for 1 (1W), 2 (2W), and 3 weeks (3W) to induce MaS. Increasing time on the MCD diet resulted in three progressive levels of steatosis: mild (<10% MaS), moderate (80% MaS), and severe (>90% MaS) (Fig 1B) with significant differences in macrosteatosis observed for animals fed for 2 and 3 weeks (p<0.001). Liver triglycerides concentration increased as MaS progressed with a significant increase (p<0.03) in animals with severe MaS (Fig 1B, 3W). Serum ALT concentration increased (p<0.001) only in animals fed MCD diet for 3 weeks (Fig 1B, 3W).

**Fig 2:**
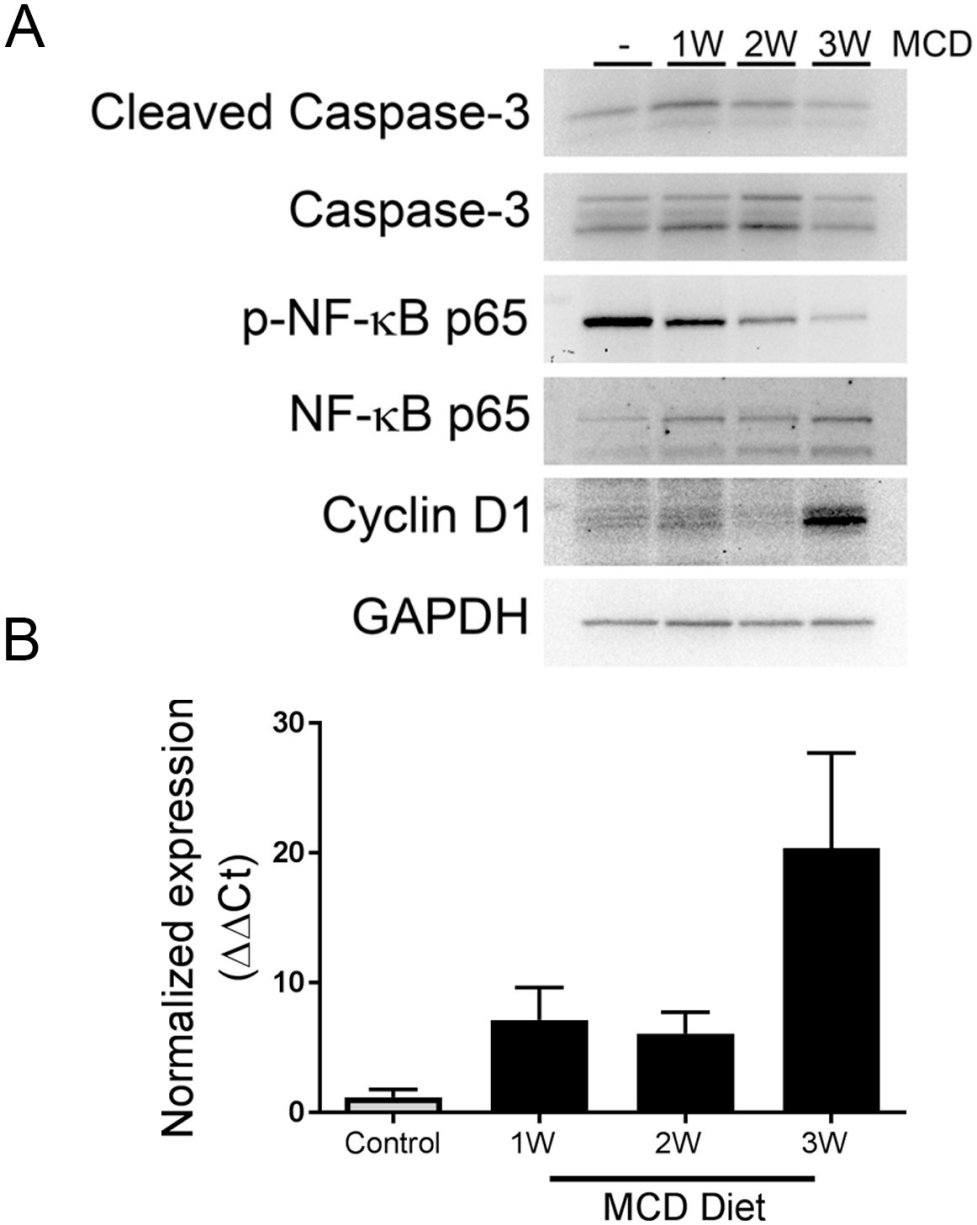
Rat model of diet-induced steatosis-driven biomarker expression. (A) Liver protein expression of molecular biomarkers in simple steatosis model. Representative immunoblots are shown with equal amounts of liver lysate analyzed. (B) Normalized expression of CCND1 in RNA extracted from liver tissue.

Rising ALTs during steatosis development led to the examination of alarmin mediators and activation of pro-survival and proliferation pathways. Liver IL-33 significantly decreased (p<0.02) in severely macrosteatotic livers (Fig 1C, 3W) while serum IL-1α significantly increased (p<0.05) in severely macrosteatotic animals (Fig 1C, 3W). There were no differences in liver high-mobility group box 1 (HMGB1) as MaS increased (Fig 1C). Activation of the pro-survival complex NF-κB p65 was found to decrease as MaS increased in the liver (Fig 2A) but without concomitant increase in activated caspase-3. Surprisingly, a strong increase in Cyclin D1 protein was observed in severely macrosteatotic livers (Fig 2A, 3W) while increased mRNA (CCND1) expression was observed in all MCD diet fed animals, with highest expression in severely macrosteatotic animals (Fig 2B).

### Simple steatosis and biomarker expression resolves upon diet withdrawal

**Fig 3:**
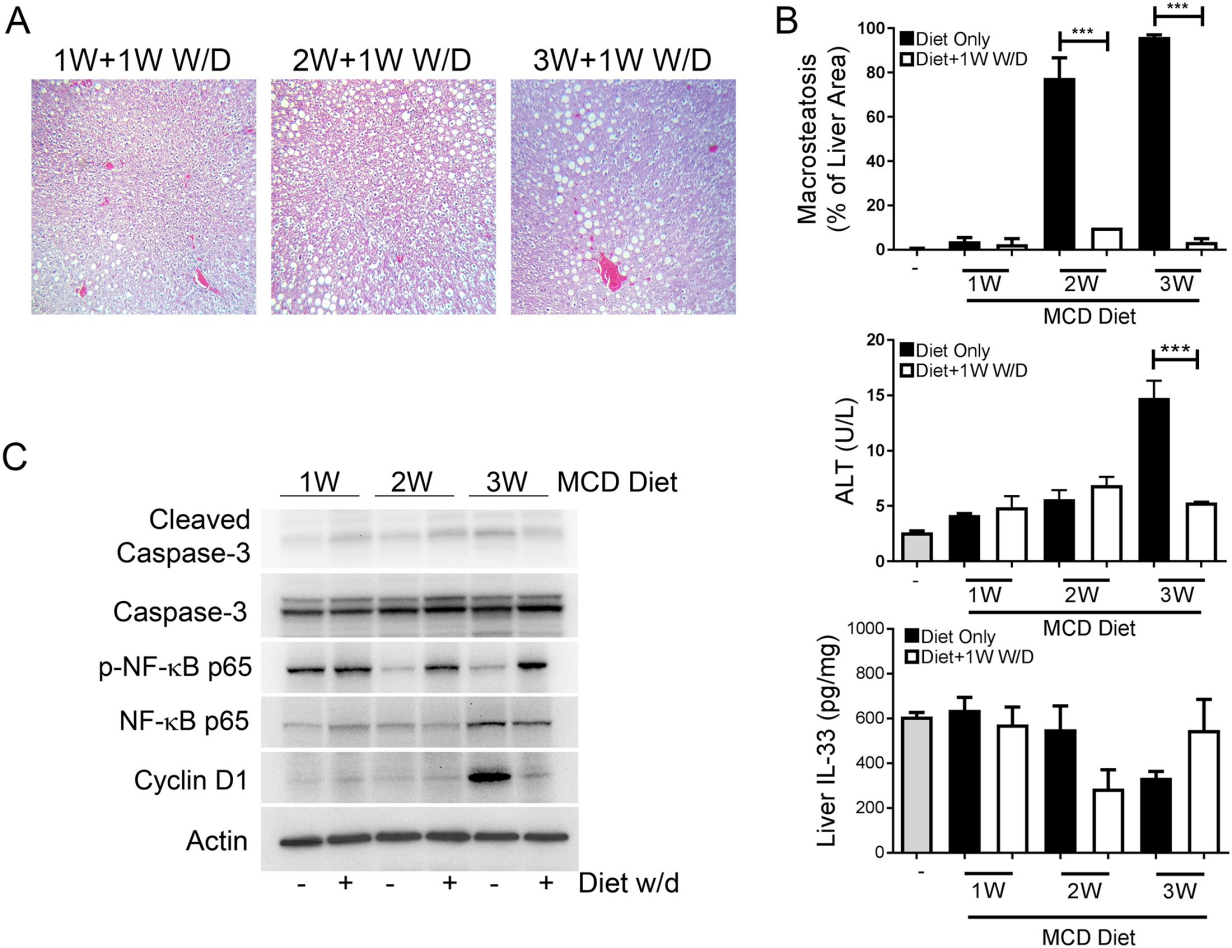
Simple steatosis and biomarker expression begins to resolve upon diet withdrawal for 7 days. (A) Trichrome stains of representative rats on MCD diet for 1, 2, and 3 weeks then withdrawn to control chow for 1 week. (B) Percentage of MaS, serum ALT (***p<0.001), and liver IL-33 levels on diet (black bars) and after diet withdrawal for 1 week (empty bars). (C) Liver protein expression of biomarker panel after one week diet withdrawal from MCD diet. Representative immunoblots are shown with equal amounts of liver lysate analyzed.

When experimental NAFLD diets are withdrawn and normal chow provided, liver steatosis resolved. To ensure the biomarkers were steatosis-driven, we withdrew the MCD diet to normal chow and monitored short-term (24 hours) and long-term (7 day) changes in expression as steatosis resolved. After 24 hours of MCD withdrawal, MaS had not resolved in animals with moderate and severe MaS, however after 7 days after MCD diet withdrawal, MaS decreased to control levels in all animals (Fig 3A and 3B). Interestingly, serum ALT significantly decreased (Fig 3B, p<0.001) with liver IL-33 returning to baseline following one week of diet withdrawal (Fig 3B). After 7 days of diet withdrawal, NF-κB p65 phosphorylation was restored and reached baseline levels after diet withdrawal (Fig 3C). No changes in activated caspase-3 were observed upon diet withdrawal for 7 days. Cyclin D1 protein returned to baseline by 7 days (Fig 3C, 3W).

### Severe macrosteatosis decreases survival post-I/R-PHx in simple steatosis model

**Fig 4:**
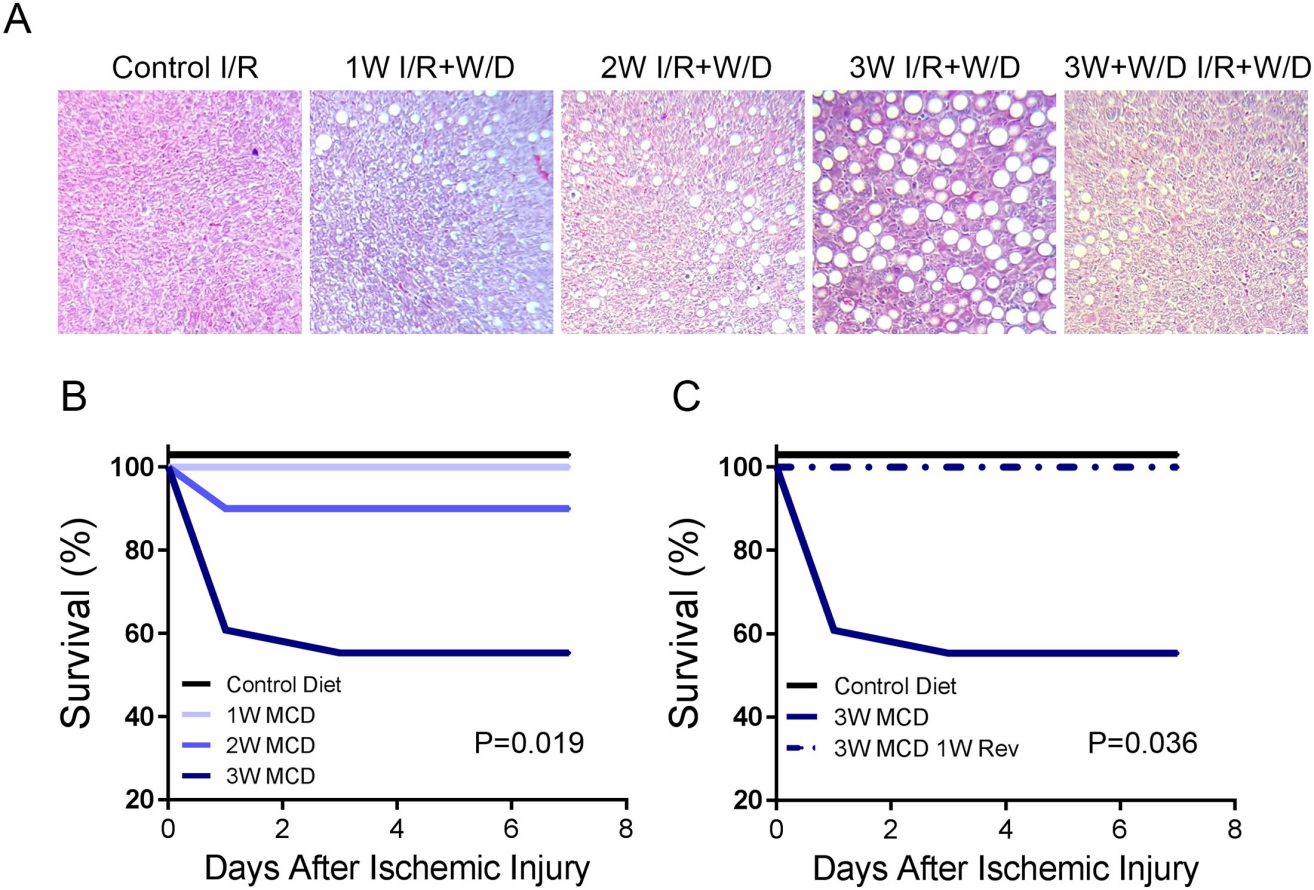
Ischemia/reperfusion and partial hepatectomy injury in simple steatosis model decreases survival. (A) Trichrome stains of representative rats fed the MCD diet for 1, 2, and 3W, underwent I/R-PHx, then fed normal diet for 1 week and survived. Rats that were fed the MCD diet for 3 weeks, withdrawn for 1 week, and survived I/R-PHx injury for 1 week on normal diet are shown as 3W+W/D I/R+W/D. (B) Survival curve of rats fed the MCD diet for 1, 2, and 3 weeks that underwent I/R-PHx injury. (C) Rats were fed the MCD diet for 3 weeks, withdrawn to control chow for 1 week, and then underwent I/R-PHx injury. Survival was recorded for 1 week.

We next determined if shifts in steatosis-driven biomarker expression was associated with survival outcomes after I/R-PHx. The I/R-PHx injury employed was tolerated without complication in animal controls and animals with mild steatosis. Residual macrosteatosis was observed while minor microsteatosis was found in 1 and 2 week MCD fed animals (Fig 4A). A slight decrease in survival (10%) was observed in animals with moderate MaS (Fig 4B, 2W) while a dramatic decrease in survival (40%) was observed in animals with severe MaS (Fig 4B, 3W) after I/R-PHx. To confirm the link between pre-injury steatosis and post-surgery survival outcomes, animals were fed the MCD diet for 3 weeks, withdrawn back to control chow for 7 days, then subjected to I/R-PHx and monitored for 7 days. As predicted, withdrawal from MCD diet resolved MaS (Fig 4A, 3W Reversal I/R-PHx), and the I/R-PHx injury was well tolerated (Fig 4C, 3W MCD 1W Rev).

### Steatosis-driven biomarker expression rebounds immediately following I/R in simple steatosis model

**Fig 5:**
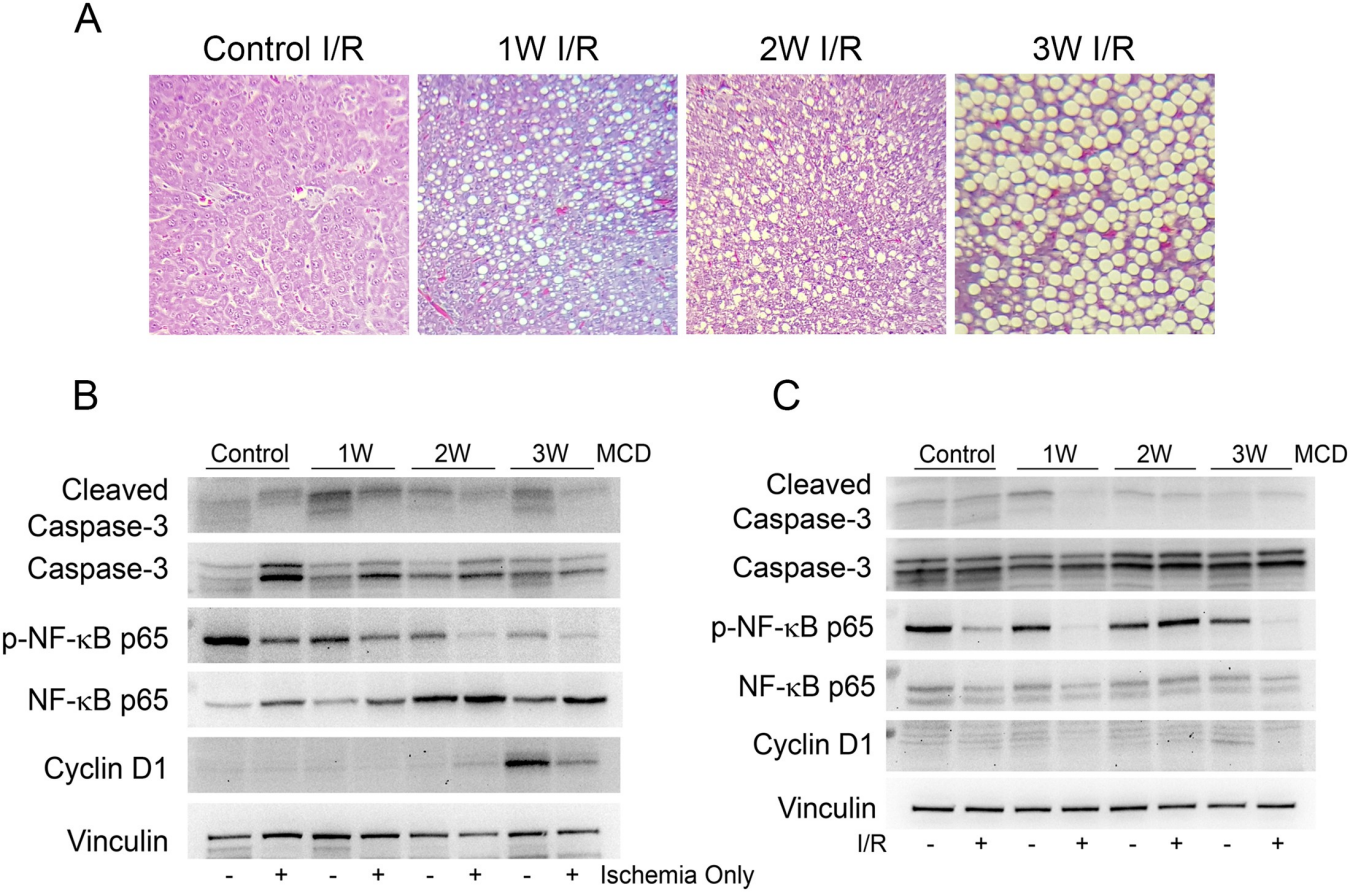
Biomarker expression immediately following ischemia and 24 hours after I/R-PHx injury in simple steatosis model. (A) Trichrome stains of control and MCD-fed rats that underwent I/R-PHx injury and withdrawn to control chow until 24 hours end point. (B) Liver protein expression of biomarker panel on liver lysate from rats fed control and MCD diets with (+) and without (-) 60 minutes of ischemia. (C) Liver protein expression of biomarker panel on liver lysate from rats fed control and MCD diets with (+) and without (-) I/R-PHx and reverted back on control chow until 24 hours end point. Representative immunoblots are shown with equal amounts of liver lysate analyzed.

To determine if ischemia impacts expression of the steatosis-driven biomarkers, animals with progressive steatosis following feeding with MCD diet were subjected to ischemia for 60 minutes. Immediately following ischemia, a decrease in phosphorylated NF-κB p65 and increase in total NF-κB p65 was observed in control and MCD fed animals (Fig 5B). Cyclin D1 protein expression immediately decreased after 60 minutes of ischemia (Fig 5B). Animals placed on MCD diet for 1–3 weeks then withdrawn to normal chow for 24 hours were compared to animals fed MCD diet for same duration that then underwent I/R-PHx prior to withdrawal to normal chow for 24 hours. MaS did not resolve after 24 hours on control chow (Fig 5A). Levels of phosphorylated NF-κB p65 and caspase-3 did not differ between the two groups (Fig 5C). A previously observed, Cyclin D1 protein expression returned to control levels 24 hours after diet withdrawal without any effect of I/R-PHx injury (Fig 5C).

**Fig 6:**
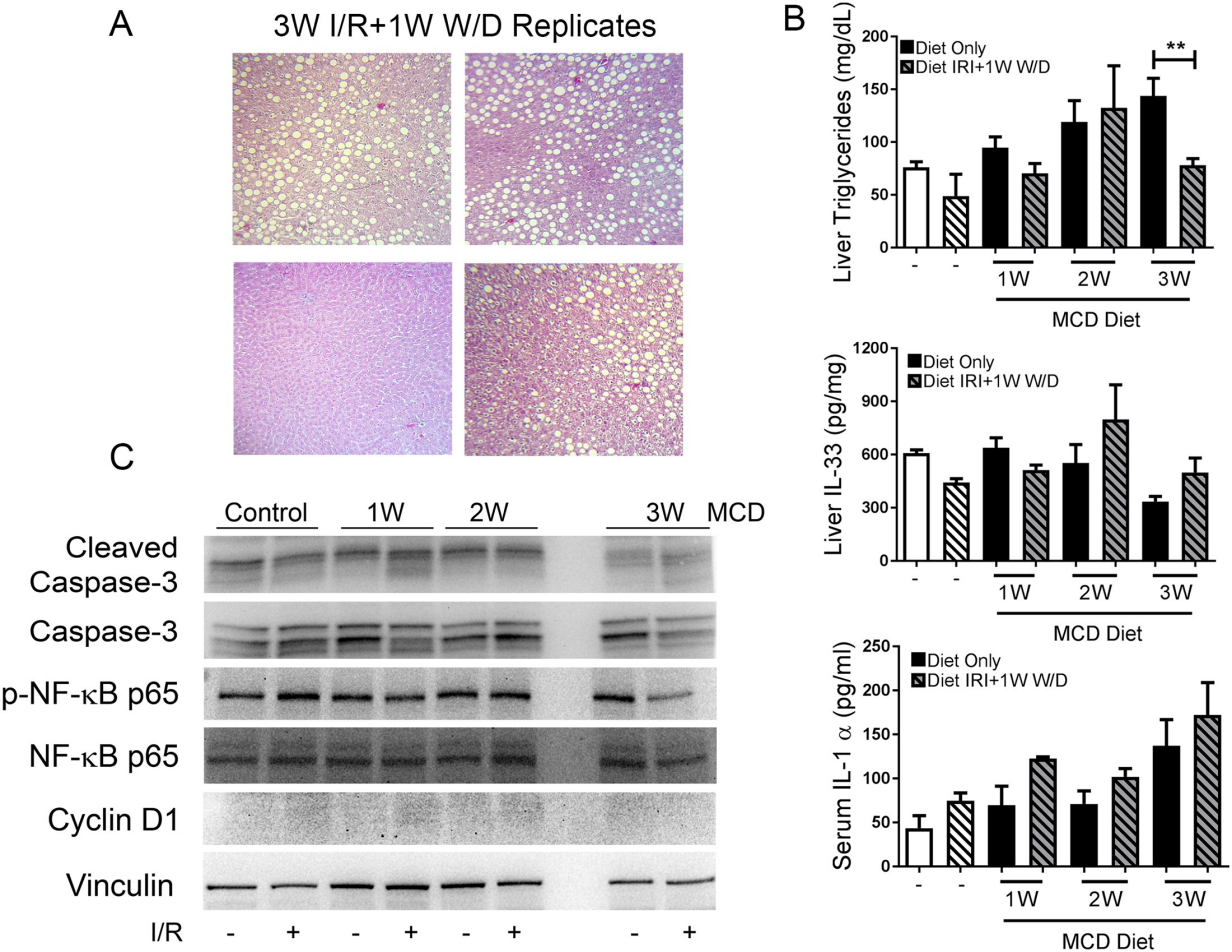
Biomarker expression resolves following I/R-PHx injury and 1 week diet withdrawal in simple steatosis model. (A) Trichrome stains of replicate rats fed MCD diet for 3 weeks, underwent I/R-PHx, and then withdrawn to control diet for 1 week that survived. (B) Liver TG (**p<0.007), IL-33 levels, and serum IL-1α levels in MCD fed rats with (hash bars) and without (filled bars) I/R-PHx. Animals that underwent I/R-PHx had diet withdrawal for 1 week. (C) Liver protein expression of biomarker panel on liver lysate from rats fed control and MCD diets with (+) and without (-) I/R-PHx and reverted back on control diet until 1 week end point. Representative immunoblots are shown with equal amounts of liver lysate analyzed.

Residual steatosis was observed in some survivors with severe MaS post I/R-PHx and return to control chow for 1 week (Fig 6A). Triglyceride levels remained stable 7 days after I/R-PHx injury and withdrawal from MCD diet to control chow (Fig 6B) except for a significant decrease at 3W (p<0.01). Liver IL-33 and serum IL-1α levels were not significantly different than post- I/R-PHx injury in either the control or MCD fed animals. Activated NF-κB p65, and Cyclin D1 protein was similar to controls in all MCD fed animals post- I/R-PHx followed by 1 week diet withdrawal to control chow (Fig 6C).

### Steatosis-driven biomarker expression prior to I/R-PHx in steatosis model predicts liver damage

**Fig 7:**
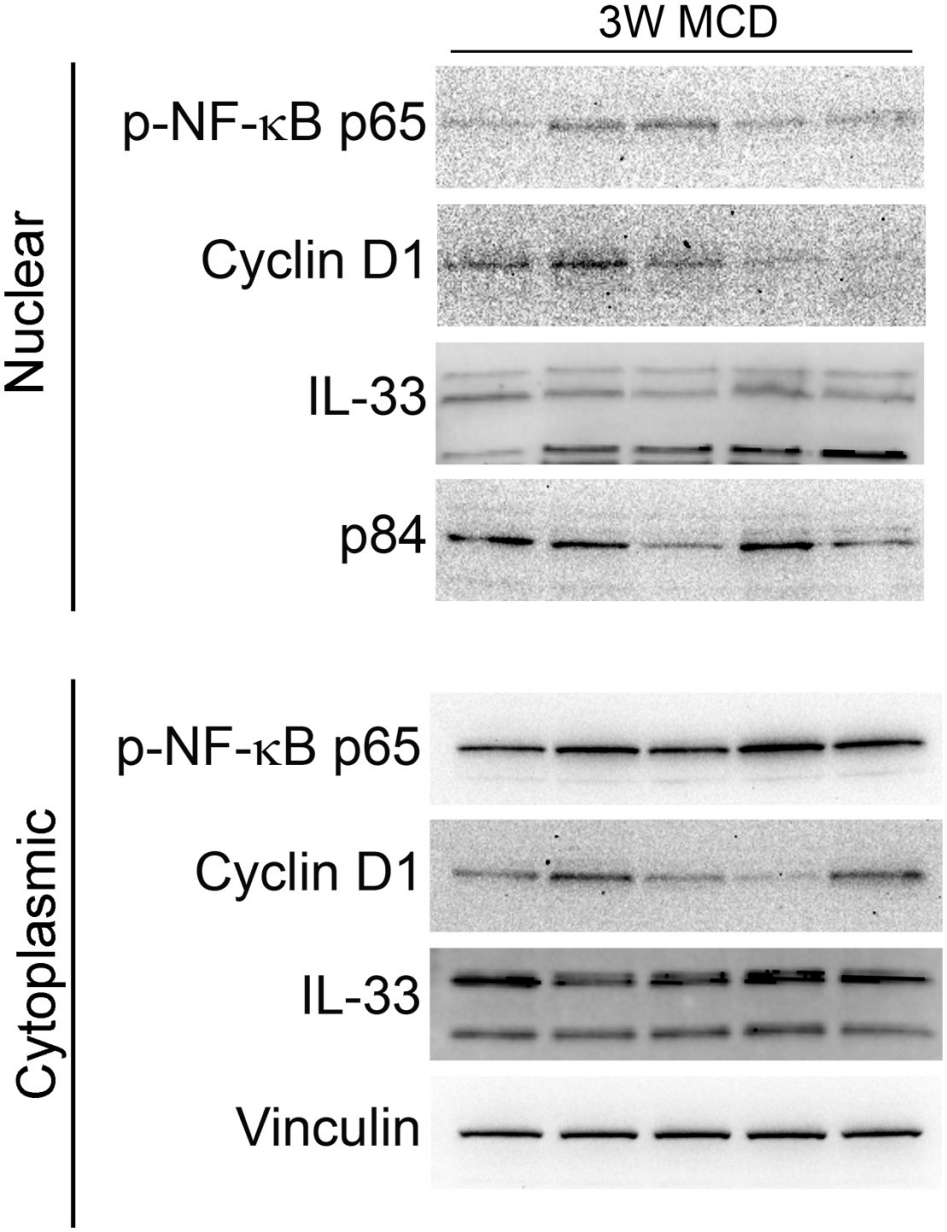
Biomarker panel predicts liver failure following I/R-PHx injury in simple steatosis model. Nuclear and cytoplasmic fractions from liver biopsies taken from rats fed MCD diet for 3 weeks. Representative immunoblots are shown with equal amounts of liver lysate analyzed.

In order to predict severe liver damage and delayed recovery following ischemia-reperfusion injury, a biopsy was performed and immediately processed to determine biomarker expression. Animals with severe MaS fell into three groupings based on outcome post I/R-PHx: non-survivors, animals with extended surgical recovery periods (>4 hours), and animals that recovered within one hour of procedure. Trichrome stains 1 week after I/R-PHx on surviving animals revealed MaS completely resolved in only one animal (Fig 6A). Biopsies prior to I/R-PHx revealed expression of phosphorylated NF-κB p65 differed among severely macrosteatotic animals with the lowest expression in the animal that did not survive the injury (Fig 7). Expression and location of Cyclin D1 also varied with non-surviving and extended recovery animals having higher expression of Cyclin D1 in the nucleus. Interestingly, the animal with complete MaS resolution one week after I/R-PHx had undetectable nuclear Cyclin D1 with expression exclusively in the cytoplasm (Fig 7). Full-length IL-33 was found to be expressed in the nucleus and cytoplasm of all groups, however cleaved liver IL-33 in the nuclear was lowest in Group 1 (Fig 7).

### Clinical validation of Cyclin D1 expression in donor liver grafts

**Fig 8:**
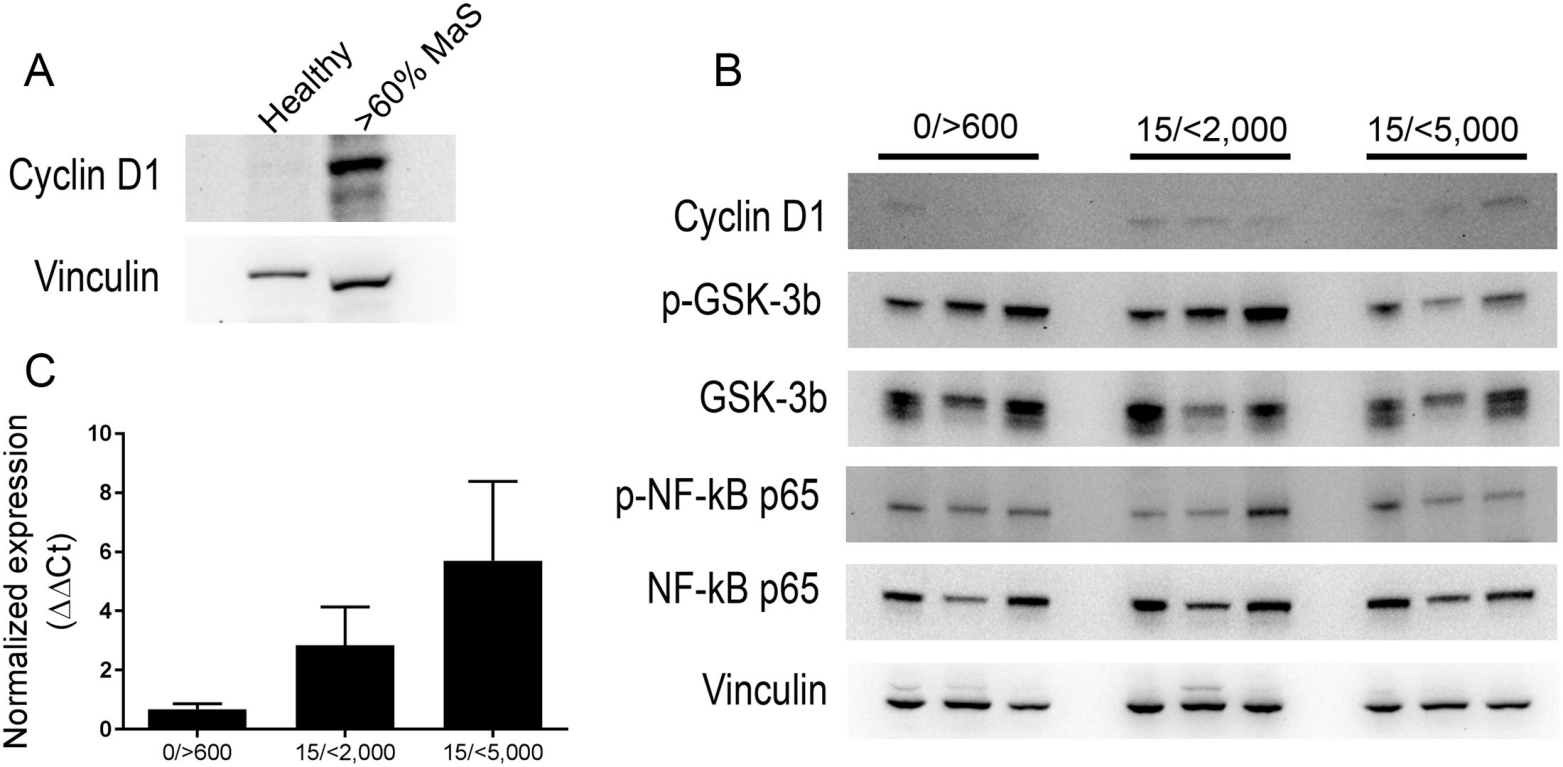
Biomarker panel in human donor liver mimics animal model. (A) Liver protein expression of Cyclin D1 in biopsies from a healthy liver and an unsuitable for transplant steatotic donor liver. (B) Liver protein expression in biopsies from donor liver grafts used for transplantation. Equal amounts of liver lysate were analyzed by immunoblotting. (C) Normalized expression of CCND1 in RNA extracted from donor liver tissue used for transplantation.

A donor liver deemed unsuitable for transplantation due to greater than 60% macrosteatosis displayed high Cyclin D1 expression compared to a healthy liver (Fig 8A). To test the biomarker panel on steatotic donor livers used for transplant, biopsies from nine donor livers were obtained from our institution. Table 1 shows the demographics of the donor and the percentages of micro- and macrosteatosis. Donor grafts were separated into three groups based on macrosteatosis percentage and recipient recovery measured by post-operative day 1 (PO D1) AST levels (Table 1). Donor grafts 10–15% macrosteatosis and the highest AST levels PO D1 corresponded with higher Cyclin D1 protein and mRNA expression compared to grafts with lower AST PO D1 levels (Fig 8B and 8C, 15/<5,000). The increase in Cyclin D1 was accompanied by a decrease in phosphorylation of GSK-3β despite differences in expression of total GSK-3β (Fig 8B). Activated NF-κB was elevated in two grafts, from 15/<2,000 and 15/<5,000, both with 10% macrosteatosis (Fig 8B).

**Table 1:** Demographics for donor liver grafts and stratification. Groups were stratified based on percentage of MaS and recipient recovery as determined by recipient AST levels one day after transplantation (post-operative day 1).

**Characteristics**		**Donor (n = 9)**
Donor age, yrs (range)		50 (26–57)
Weight (kg, range)		90 (62–163)
BMI		29 (24–54)
**Groups**	**MaS [%]**	**AST PO Day 1**
Group 1	0	>600
Group 2	10–15	<2,000
Group 3	10–15	<5,000

## Discussion

The increasing incidence of NAFLD, now estimated to be 20–30% of the population[8], will begin to reflect in the donor liver pool. Despite elevated PNF risk, grafts with moderate to severe MaS [4, 9] have been successfully transplanted[10]. This suggests the biology driving PNF risk is not solely dependent on the percentage of macrosteatosis and may involve patient specific responses to lipid-induced stress. In this study, we developed an animal model using the MCD diet for transplant relevant simple steatosis. Our data suggests a steatosis-driven biomarker may help predict severe liver failure following I/R injury.

I/R injury in animal models of NASH and cirrhosis have shown an increased mortality risk precluding the use of these organs for orthotopic liver transplantation[11]. There are limited models of simple steatosis that provide insight into PNF risk. The MCD diet was chosen as this diet more closely resembled donor steatosis in contrast to Western diet that resulted in hepatocyte ballooning. To study risk associated with simple steatosis, feeding with the MCD diet was reduced to 3 weeks as NASH progression occurs after 5 weeks on the diet[12, 13]. MCD feeding for 2 weeks was sufficient to yield transplant-relevant simple steatosis in the absence of inflammation and fibrosis. Animals fed the MCD diet for 2 and 3 weeks did not show differences in percentage of MaS, however lipid droplet size, liver triglycerides, and serum ALT progressed. These changes indicate hepatocytes reached a lipid accumulation threshold resulting in injury, unregulated cell death, and alarmin release.

As confirmation of mild hepatocellular damage and alarmin release, we found increased serum IL-1α and decreased liver IL-33 in rats with severe MaS. IL-33, and IL-1α are released after hepatocytes undergo cellular injury or necrosis[14] as seen in NASH rodent models[15]. Undetectable levels of serum IL-33 and stable liver HMGB1 suggests the level of hepatocyte damage in our simple steatosis model is not severe enough to result in systemic release of these alarmins into the serum. We also did not observe nuclear signatures of apoptosis histopathologically in the liver or by analyzing activation fragments of caspase-3 via immunoblot as observed in NASH models[16].

The lack of inflammatory cytokines as steatosis progressed correlated with decreasing activation of NF-κB p65, a transcriptional activator of TNF-α and IL-6. This was also observed in mice fed the MCD diet for 5 weeks[12]. Mice treated with IL-33 prior to I/R injury had reduced serum ALT release suggesting a hepatoprotective role[17]. A recent study showed increased liver IL-33 expression in diet-induced NASH mice[18]. NASH model using high-fat diet results in hepatocyte ballooning as opposed to simple steatosis model used in this study. An association between IL-33 and NF-κB has been proposed[19], but whether this interaction is positive or regulatory remains controversial[19, 20]. Our results demonstrate steatosis-induced hepatocellular damage marked by ALT/IL-1αrelease and shifts in the form of decreased activation of NF-κB and IL-33 aimed at reducing injury.

Hepatocytes alter their molecular machinery to deal with lipid induced stress. Cyclin D1 has regulatory functions in downregulating lipogenesis[21] and is elevated in rodents with NASH[22]. Cyclin D1 represses the transcriptional activation activities of several proteins involved in lipogenesis through direct binding[21, 23, 24]. Cyclin D1 was elevated in animals with severe MaS despite overnight fasting which is sufficient to drive Cyclin D1 expression to baseline levels in hepatocytes[25]. How lipid accumulation contributes to increased expression of Cyclin D1 remains under investigation, however may involve Cyclin D1’s function in downregulating genes involved in lipogenesis.

The stress induced by lipid accumulation resolves once the lipids are removed. Both steatosis and lipid-induced hepatocellular injury resolves within weeks after transplantation in grafts accompanied by dietary restrictions on the recipient[4, 26, 27]. In our simple steatosis model, signals of hepatic injury and lipid-driven molecular biomarkers resolved to baseline following return to control chow. Similarly, mice fed MCD diet for 8 and 16 weeks then withdrawn to control chow for 2 weeks showed baseline serum ALT levels and steatosis[28] supporting our results. These data indicate once the steatosis-driven stress is removed and resolved, so does the hepatic injury and damage.

The molecular biomarkers designate which animals have predisposed hepatocellular injury with the same percentage of macrosteatosis. To determine how these animals would response to a second hit, steatotic animals were exposed to I/R-PHx injury. The decreased survival in animals with severe MaS demonstrates the combination of lipid-induced accretion of injury, molecular pathway disarray, and secondary injury in the form of I/R-PHx can causes significant liver failure. However, some animals with severe MaS did survive indicating molecular biomarker expression was not the same in all animals.

In animals with severe MaS, a biopsy was performed to assess steatosis-driven biomarker expression and location to predict survival post-I/R-PHx injury. Animals with decreased nuclear IL-33 and activated cytoplasmic NF-κB resulted in death or prolonged recovery post-injury. These changes likely caused loss of hepatoprotective function by IL-33 and delayed activation of pro-survival genes by NF-κB. Increased Cyclin D1 expression in the nucleus correlated with worse outcomes after I/R-PHx injury. Cyclin D1 represses the transcription activators hepatocyte nuclear factor-4 alpha and peroxisome proliferator-activated receptor-gamma involved in lipogenesis through direct binding[21, 29]. Under the stress of lipid accumulation, Cyclin D1 expression may be increased and remain sequestered in the nucleus possibly bound to proteins involved in lipogenesis. This prevents Cyclin D1 from initiating cell cycle progression resulting in severe liver failure or delayed recovery.

Safe utilization of steatotic donor livers for transplantation differ among centers with grafts <30% MaS being transplanted and >30% discarded[26, 30]. In an unsuitable for transplant steatotic donor liver (>30% MaS), high Cyclin D1 expression was confirmed. Steatotic grafts positively correlate with increased AST levels in recipients 24 hours after transplantation[31]. As such, a pilot clinical subset of selected steatotic donor livers with delayed graft function was used to confirm results observed in our rat model. A similar trend was seen in the clinical subset with an overall decrease in activated NF-κB and increased Cyclin D1 mRNA expression. Proliferative role of Cyclin D1 was ruled out due to the absence of phosphorylated Rb and activated GSK-3β suggesting Cyclin D1 function in steatotic grafts may be similar to that observed in our simple steatosis animal model. Alterations in the molecular pathways investigated in this study during the development and persistent steatosis may develop more liver injury and PNF risk post-transplantation.

The growing discrepancy between liver transplant candidates and available donor livers has increased utilization of high DRI livers and necessitates a more thorough understanding the mechanisms increasing PNF risk with these organs. Developing risk biomarkers will be challenging given the differences in molecular targets among these unique DRI categories. While treatment options for patients with NAFLD remain focused on life-style changes which include dietary restrictions in calories and fats[32], these options remain dependent on the patient. This study provides the first rodent model for diet-induced simple steatosis to study PNF after I/R-PHx injury. We have identified three potential biomarkers, IL-33, Cyclin D1, and NF-κB whose expressions are altered in severely steatotic livers at risk for severe liver damage post I/R-PHx. There markers may provide insight in potential risk of PNF resulting in safer utilization of these steatotic grafts.
